# Rapid Growth of Pelvic Cyst during Pregnancy: A Case Report

**DOI:** 10.1155/2019/3120921

**Published:** 2019-05-13

**Authors:** Yoko Fujimoto, Hironori Takahashi, Kenji Horie, Takeo Nakaya, Toshiro Niki, Hiroyuki Fujiwara, Shigeki Matsubara

**Affiliations:** ^1^Department of Obstetrics and Gynecology, Jichi Medical University, Japan; ^2^Pathology, Jichi Medical University, Japan

## Abstract

We describe a patient with bilateral cystic tumors of the pelvis. The left one rapidly grew during pregnancy and combined with the right one, whose clinical course made diagnosis difficult. A pregnant woman with a history of laparotomy was referred to us due to suspected bilateral pelvic cysts. The left-sided cyst had rapidly grown to 27 cm in diameter and merged with the right cyst, forming a large cyst occupying the entire pelvic cavity in the third trimester. Considering this rapid growth, cesarean section and resection of the cyst were performed at 37th week. The resected cyst consisted of two components: a large unilocular cyst containing serous fluid and a multilocular cyst suggestive of ovarian mucinous cystadenoma in the right ovary. The wall of the former largely lacked lining epithelium, but it was partly continuous with the latter mucinous epithelium. Immunohistochemically, estrogen and progesterone receptors were focally positive in the cyst wall, suggesting that pregnancy-associated sex-hormones may have contributed to the rapid growth of the cyst. We diagnosed this condition as a peritoneal inclusion cyst margining with a right ovarian mucinous cystadenoma. Peritoneal inclusion cyst should be considered in the differential diagnosis of a rapidly growing pelvic mass during pregnancy.

## 1. Introduction

Large cystic tumors of the pelvis during pregnancy are rarely observed [1]. Treatment of large cystic tumors of the pelvis during pregnancy is challenging. Rapidly growing cysts with images and serum-markers indicative of malignant ovarian tumors usually prompt obstetricians/gynecologists to perform surgery during pregnancy, whereas solely rapid growth may make them hesitate to do so since surgery during pregnancy frequently results in preterm delivery [2].

An inclusion cyst refers to serous fluid inclusion in the enclosed peritoneal cavity. Laparotomy, endometriosis, and abdominal infection have been reported as causes of this cyst [3–5]. The cysts occur most frequently in patients younger than 50 years of age (92%; 23/25) [6]. In addition, a gonadotropin-releasing hormone agonist and oral contraceptives were reported to decrease their size [3, 7–9], suggesting that sex-hormones may promote fluid secretion, thereby enlarging the inclusion cyst [3, 7, 10]. To our knowledge, there have been only a few reports of inclusion cysts during pregnancy [4, 11]. Moreover, there has been no previous report of a rapidly growing pelvic inclusion cyst during pregnancy.

We experienced a case of rapidly growing pelvic cystic tumor during pregnancy. We speculate that the peritoneal inclusion cyst had grown rapidly during pregnancy under pregnancy-associated sex-hormones.

## 2. Case Report

**Figure 1:**
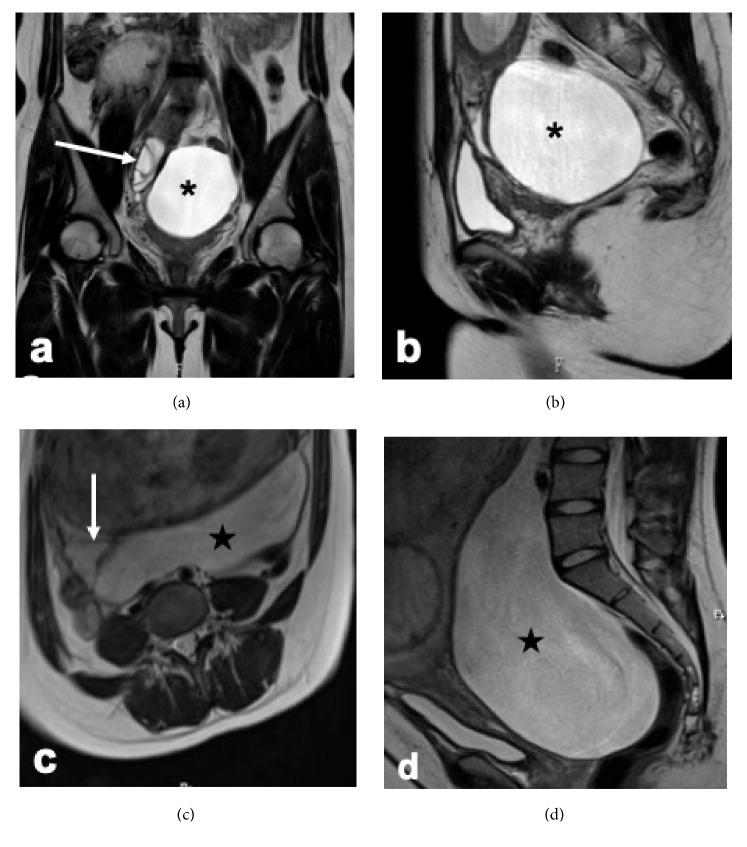
T2-weighed magnetic resonance imaging at 9 (a, b) and 32 (c, d) weeks of gestation. (a) Bilateral pelvic masses with a horizontal view. A right-sided multilocular mass (arrow) and left-sided unilocular mass (asterisk) are shown. We suspected bilateral ovarian tumors at this point. (b) Sagittal view. The left-sided unilocular mass (asterisk) is shown. (c) Horizontal view. The right-sided multilocular mass (arrow) is involved in or at least is located very close to the large mass (star). (d) Sagittal view. The left-sided mass (star) occupies the pelvic cavity.

A 24-year-old pregnant woman (G2P1) was referred to us due to suspected bilateral ovarian cysts at 8 weeks of gestation. She had undergone ovarian cystectomy twice under open surgery: left and right ovarian cystectomy for mature cystic teratoma and mucinous cystadenoma, respectively. She had no additional medical history or familial medical history. Transvaginal ultrasound and magnetic resonance imaging (MRI) (Figures 1(a) and 1(b)) revealed two pelvic cysts. The left-sided unilocular cyst was 9 cm in diameter. The right-sided multilocular cyst was 5 cm in diameter. We diagnosed this condition as bilateral ovarian cysts.

Although the serum levels of tumor markers (CA125, CA19-9, and CEA) were normal for a pregnant woman, considering the large size of the cyst, cyst resection was attempted at 14 weeks; however, it was converted to probe laparotomy. Marked adhesion around the cysts, posterior uterus, and Douglas' pouch made cyst resection impossible as extensive adhesiolysis may cause uterine damage and also uterine contractions after surgery. Gross examinations revealed no metastatic lesions or lymph node swelling. Abdominal fluid cytology revealed no malignant cells.

At 32 weeks of gestation, MRI revealed that the left-sided cyst size had increased to 27 cm in diameter (Figures 1(c) and 1(d)), although she was asymptomatic. As shown in Figure 1(c), the right-sided multilocular cyst became very close to the left monocytic cyst. At this stage, the left large monocytic cyst appeared to merge with the smaller right multilocular cyst, forming a large cyst occupying the entire pelvic cavity, which was later confirmed by laparoscopic findings.

This large cyst showed no solid-part or papillary growth. The serum levels of tumor markers remained normal. Malignant ovarian tumor could not be ruled out but was considered less likely. We weighed merits and demerits between relaparotomy for tumor resection during pregnancy and a wait-and-see approach for several weeks; the former is likely to require extensive adhesiolysis and may cause preterm delivery. We decided on the latter strategy, since resection should be performed in the event of a size increase or images indicative of malignancy. The fetus normally developed without fetal growth restriction.

**Figure 2:**
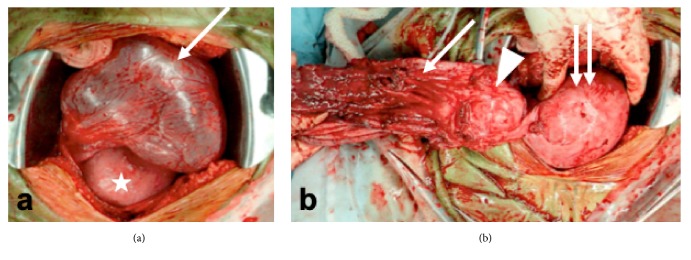
Intra-operative findings during cesarean section (CS) and tumor resection at 37 weeks of gestation. (a) The large cyst (arrow) is on the dorsal and caudal sides of the uterus (star) following CS. (b) The large cyst wall (arrow) is adjacent to the right multicystic ovarian tumor (arrowhead). We resected the right adnexa and a part of the cyst wall. The wall of the large cyst was coarse and weak, suggestive of degeneration. Uterus (double arrows).

**Figure 3:**
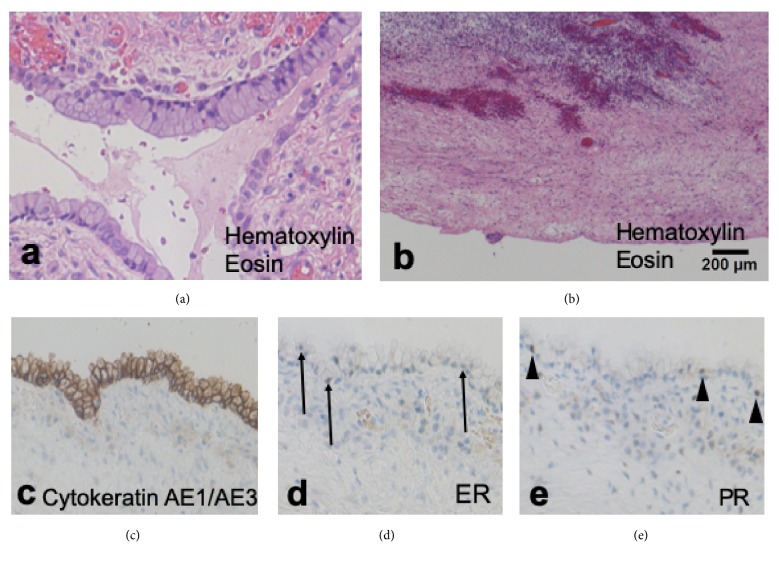
Histological findings of the right ovarian tumor (a-d). (a) The epithelium of the right ovarian tumor without dysplasia contains mucin. It is consistent with mucinous cystadenoma (Hematoxylin and Eosin stain, x20). (b) The large cyst wall containing serous fluid shows partial defects of epithelium. This is due to adhesion of the abdominal cavity and cyst. (c-e) The large cyst wall is strongly positive, partly weakly positive (arrows), and partly positive (arrowheads) for cytokeratin AE1/AE3, estrogen receptors (ER), and progesterone receptors (PR), respectively (x20).

Cesarean section and tumor resection were performed at 37+4 weeks of gestation, yielding 3,012-g male infant with Apgar score 8/9 at 1/5 minutes, respectively. The infant did not have congenital abnormalities. After the completion of cesarean section, we ruptured the wall of this large cyst, with care to avoid the cyst content entering into the abdominal cavity. A large amount of serous fluid was drained. This large cyst was a multicystic cyst (5 cm), considered to be the right multicystic ovarian cyst that had been observed from the first trimester. The wall of the large cyst showed marked adhesion to the peripheral peritoneal cavity. We resected it as widely as possible together with right salpingo-oophorectomy (Figures 2(a) and 2(b)). The left ovary was macroscopically normal, and thus there was no evidence of the left ovarian tumor. The resected tumor consisted of a large unilocular cyst with serous fluid and a mucinous cystadenoma (Figures 3(a) and 3(c)). In the former, lining epithelium was absent in many parts (Figure 3(b)) and mucinous epithelium was occasionally found in continuity with the cyst wall of the latter (right ovarian cystadenoma). No malignant cells were found in the resected specimen. Immunohistochemistry revealed focally positive staining for estrogen and progesterone receptors on the resected cyst wall (Figures 3(d) and 3(e)). At 12 months after the delivery, left ovary remained normal and the retention cyst did not recur. An informed consent for this reporting was obtained from this patient.

## 3. Discussion

Here, we report bilateral pelvic cysts, in which the left one grew rapidly during pregnancy and merged with the right cystic tumor. Histologic examination revealed a large cyst containing serous fluid and a mucinous cystadenoma, with both showing no malignant cells. To explain the clinical course and histogenesis of this tumor, we speculated as follows: originally, there was a left serous cyst (9 cm) (possibly an inclusion cyst) and a right ovarian mucinous cystadenoma (5 cm). The former inclusion cyst merged with the latter (a mucinous cystadenoma), resulting in this unique composite tumor.

**Figure 4:**
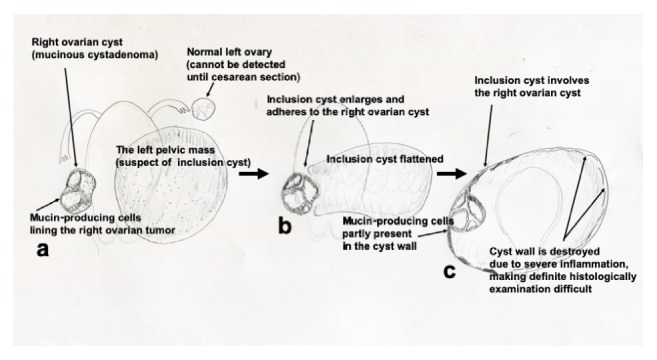
Schematic presentation of enlargement of the inclusion cyst. (a) At 9 weeks, the right mucinous cystadenoma and left cyst (later diagnosed as an inclusion cyst) existed independently. (b) During gestation progressing, the inclusion cyst became larger. The right ovarian cyst and inclusion cyst became close. (c) Finally, the right ovarian cyst was involved in the inclusion cyst. Mucin-producing cells were observed mainly on the near-side of the right ovarian cyst. The wall of the inclusion cyst was largely destroyed due to inflammation, which made it difficult to conduct a detailed histological examination such as the confirmation of mesothelial cells in the inclusion cyst wall.

Inclusion cysts can be formed as follows: an enclosed cavity is formed following abdominal or pelvic surgery or its inflammation, fluid is secreted from the ovarian surface or cyst wall, and fluid production increases, exceeding its absorption, leading to a large fluid-containing cyst [3–7, 9, 10]. In the present case, an inclusion cyst may have developed in a space created by the previous two surgeries. This cyst, having been 9 cm in diameter in the first trimester, increased in size with the progression of gestation, possibly related to increased sex-hormone secretion during pregnancy. The wall of this inclusion cyst was positive for estrogen and progesterone receptors, and, thus, fluid production may have increased. During cyst progression, it may have involved the preexisting right ovarian mucinous cystadenoma. Thus, this resulted in a composite tumor consisting of a large serous cyst (inclusion cyst) and a mucinous cystadenoma (right ovary) (Figure 4). The ovarian surface epithelium, developing in an inclusion cyst, may have also secreted fluid into the closed cavity of the inclusion cyst. This progression may explain the present clinical course.

One inconsistency with this proposed scenario may be that we could not detect typical mesothelial cells in the cyst cavity. The cyst cavity was partly covered by mucin-producing cells. This may have resulted from mucin-producing cells being incorporated with the large inclusion cyst during the merging of the two masses. Adhesion of the large cyst wall was marked, and, thus, some of its parts could not be removed. This may have made it difficult to confirm typical mesothelial cells in the cyst wall. We believe that the lack of confirmation of histologically evident mesothelial cells in the cyst wall does not refute our suggested scenario, considering the difficulty of confirming the histological findings.

A history of multiple abdominal surgeries and an increased secretion of sex-hormones during pregnancy are not specific to this patient. We do not know why this inclusion cyst showed rapid growth. One possible reason is that laparotomy during pregnancy may have caused additional inflammation at the site, further increasing the surrounding tissue tension and increasing fluid production. However, even if this was the case, laparotomy would remain unavoidable even though it may eventually lead to probe laparotomy. Findings based on a single case cannot lead to a conclusion. However, we suggest that peritoneal inclusion cyst should be considered in the differential diagnosis of a rapidly growing pelvic mass during pregnancy, especially when the patient has a risk associated with it such as a history of multiple abdominal surgeries or laparotomy during pregnancy.
